# Modulation of autonomic activity in neurological conditions: Epilepsy and Tourette Syndrome

**DOI:** 10.3389/fnins.2015.00278

**Published:** 2015-09-17

**Authors:** Yoko Nagai

**Affiliations:** Department of Clinical Medicine, Clinical Imaging Sciences Centre, Brighton and Sussex Medical School, University of SussexBrighton, UK

**Keywords:** biofeedback, electrodermal activity, sympathetic activity, behavioral control, epilepsy, Tourette Syndrome

## Abstract

This manuscript considers the central but neglected role of the autonomic nervous system in the expression and control of seizures in epilepsy (small) and tics in Tourette Syndrome (TS). In epilepsy, consideration of autonomic involvement is typically confined to differential diagnoses (e.g., syncope), or in relation to Sudden Unexpected Death in Epilepsy (SUDEP). Investigation is more limited in Tourette Syndrome. The role of the autonomic nervous system in the generation and prevention of epileptic seizures is largely overlooked. Emotional stimuli such as anxiety and stress are potent causes of seizures and tic activity in epilepsy and TS, respectively. This manuscript will describe a possible neural mechanism by which afferent autonomic projections linked to cognition and behavior influence central thalamo-cortical regulation, which appears to be an important means for controlling both seizure and tic activity. It also summarizes the link between the integrity of the default mode network and autonomic regulation in patients with epilepsy as well as the link between impaired motor control and autonomic regulation in patients with TS. Two neurological conditions; epilepsy and TS were chosen, as seizures and tics represent parameters that can be easily measured to investigate influences of autonomic functions. The EDA biofeedback approach is anticipated to gain a strong position within the next generation of treatment for epilepsy, as a non-invasive technique with minimal side effects. This approach also takes advantage of the current practical opportunity to utilize growing digital health technology.

## Introduction

Penfield and Jasper (1954) observed autonomic responses to cerebral stimulation in humans undergoing epilepsy surgery under local anesthesia. Different cortical and subcortical sites of stimulation engendered a range of autonomic changes, suggesting that epileptic foci can also differentially influence autonomic activity. Correspondingly, focal discharges in the temporal and frontal cortices could result in autonomic disturbances affecting arterial blood pressure, heart rate and rhythm, and cardiac neural discharge. Animal studies demonstrated that even minimal epileptogenic activity (single spikes) can alter cardiac neural discharges and arrhythmias (Schraeder and Celesia, 1977). It is also noteworthy that patients with epilepsy, compared with healthy controls, show interictal autonomic abnormalities (in electrodermal activity and heart rate variability; HRV) (Drake et al., 1998; Zaatreh et al., 2003; Berilgen et al., 2004; Evrengül et al., 2005). Specifically, patients with epilepsy, compared with healthy controls have significantly higher amplitude and latency of sympathetic skin response, and consistently lower HRV (an index of vagal parasympathetic tone). The reduced HRV and impaired baroreflex sensitivity may decrease the threshold of the body's capacity to cope physiologically with seizure events (Surges et al., 2009). Sudden unexpected death associated with epilepsy (SUDEP) may be a key consequence of epilepsy-associated autonomic dysfunction (Nashef et al., 1998; Surges et al., 2009). In epilepsy and other neurological and neuropsychiatric disorders such as Tourette Syndrome, patients frequently report that emotional stress, (which typically results in enhanced sympathetic and reduced cardiac parasympathetic activity) triggers an exacerbation of symptoms (Antebi and Bird, 1993; Spector et al., 2001; Steinberg et al., 2013a,b). Moreover, some patients with epilepsy are able to suppress or terminate seizures by developing behavioral “countermeasures” that depend on intentional regulation of autonomic arousal (Fenwick, 1991). Together, these observations highlight the bidirectional coupling of central epileptogenesis with autonomic regulation wherein seizure activity can provoke potentially pathological autonomic change. On the other hand the state of peripheral autonomic arousal also impacts on seizures occurrence. This coupling may also add a psychophysiological dimension to epilepsy control by neural systems controlling emotional and bodily arousal influence seizure thresholds.

The current manuscript will present a perspective on the autonomic involvement in the generation and suppression of symptoms of two neurological conditions, epilepsy and Tourette Syndrome. These neurological conditions are the focus of the current manuscript, since the occurrence of seizures and tics are explicit measures of clinical outcome on which we can evaluate the effects of autonomic function and its modulation. A model will be discussed regarding the mechanisms of interaction between emotional triggers, autonomic regulation, and the expression of both seizures and tics.

## Emotional triggers of seizures and tics

Epilepsy is the most common serious neurological disorder, affecting 0.5–1% of the population worldwide. The prognosis of intractable epilepsy has not been changed significantly by the recent introduction of new antiepileptic drugs; one third of patients with epilepsy do not become seizure-free and therefore carry an increased risk of morbidity and mortality (Hopkins et al., 1995). Epilepsy is a neural disease classified into three types based on broad underlying mechanisms: genetic, structural/metabolic, and unknown cause (Noebels et al., 2002). The majority of genetic causes of epilepsy entail mutations in genes encoding ion channels resulting in altered neural function in the central nervous system (Noebels et al., 2002). These may well also affect metabolic functions and these same mutations very likely alter autonomic nervous system function. Tourette Syndrome is a neuropsychiatric disorder of developmental origin, characterized by involuntary motor and phonic tics. Prevalence is reported as up to 1 in 100 school children with one third of patients continuing to express significant symptoms into adulthood (Robertson, 2000). There is general agreement that TS is strongly genetic (Robertson, 2000). Studies of twins demonstrate the heritable expression of the condition, with rates significantly higher in monozygotic twin pairs (77%) compared to dizygotic twins (23%) (Price et al., 1985).

In epilepsy, it is generally considered that seizure occurrence in patients take place randomly without any apparent causes. Patients are often insecure due to the unpredictability of the occurrence of their seizures and this can markedly limit patients' social activities. However, there is much evidence that patients' seizures are often linked to fluctuations in emotional, mental and physiological states in their daily life, and that anxiety is a common trigger of seizures (Fenwick, 1991; Antebi and Bird, 1993; Spector et al., 2001; Nakken et al., 2005; Petitmengin et al., 2006; Pinikahana and Dono, 2009). This association was acknowledged well over a century ago. Gowers (1885) described, “Of all the immediate causes of seizures, the most potent are psychical-fright, excitement, and anxiety,” However, this observation has not been the focus of much scientific investigation until relatively recently. A questionnaire study of 100 patients with epilepsy from an outpatients clinic (seizure type: 56% multiple, 51% partial, 24% partial with secondary generalization and 20% generalized) found that negative mental states were a potent cause of seizures: Tension and anxiety was reported to trigger seizure occurrence and increase seizure frequency in 66% of patients, unhappiness and depression in 44%, and overexcitement in 38% of patients. Interestingly, 92% of patients were also able to recognize states that could either trigger or suppress their seizures (Antebi and Bird, 1993). Extending this finding, up to 25% of patients reported that they were actually able to induce a seizure by their own will, and most of these patients also had their own strategy (ex destraction of attention, clenching fist) to suppress their seizures (Fenwick, 1991). Another questionnaire survey of 100 epilepsy patients reported that a significant percentage (over 90%) can identify high-risk (trigger) situations, typically states of heightened stress/tension and anxiety. Low risk (safe) situations, on the other hand, seemed to be more difficult to recognize. Patients identified low risk situations as being times when they were relaxed, not feeling stressed, feeling restful, not worried (55.4%), mentally occupied, concentrating, being busy (29.2%), being happy (18.5%), and when not tired (7.7%) (Spector et al., 2001). These studies were replicated in larger clinical samples (309 and to 1677 patients) and strongly linking emotional stress, tiredness and sleep deprivation to seizure occurrence, consolidated evidence that emotional, mental and physiological states influence the expression of epilepsy (Nakken et al., 2005; Pinikahana and Dono, 2009).

An increase in the number and intensity of tics in patients with Tourette Syndrome when discussing sensitive topics or during states of physical arousal associated with significant emotional disturbance has been observed in patients (Clinical observation, if not in majority of patients). There is likely also an interaction with affective instability and related behavioral problems, since comorbid neuropsychiatric symptoms such as attention deficit hyperactivity disorder (ADHD), Autism Spectrum Disorder (ASD), mood disorder and obsessive compulsive disorder (OCD) are common (Hoekstra et al., 2013). One study used video clips to induce emotional changes in Tourette patients (Wood et al., 2003). Here, increased tics were observed during the anticipation, low-grade experience and resolution of emotional changes (Wood et al., 2003). Interestingly, fewer tics were observed during situations eliciting anger and happiness. It is reported that the intensity of concentration/attention associated with high emotional engagement may be responsible for this reduction, however further work is needed to better understand biological mechanisms as the sample size (*n* = 4) of the study was small (Wood et al., 2003). The psychological influence on the expression of tics is apparent in that the intensity of tics (or the urge to tic) is strongly influenced by a patient's belief about tics (Steinberg et al., 2013a). The self-report inventory of premonitory urges for tics was highly correlated with scale of beliefs about tic (Steinberg et al., 2013a). Tic activity is also related to stressful events occurring in daily life (Steinberg et al., 2013b).

Despite the recognition that emotional triggers influence symptom expression, the pathological mechanisms by which emotional arousal and autonomic disturbance act on neural substrates responsible for triggering of either seizures or tics are poorly understood. Behavioral effects on the seizure focus were demonstrated in non-human primates using the almina-gel model of epilepsy (Lockard and Wyler, 1979). Wyler et al. (1975) identified two types of neural composition surrounding a seizure focus: Group 1 neurons, are highly epileptogenic and constantly fire in neural clusters. This class of neuron shows non-attenuation of bursting neural activity during the operant condition (increased attention). Surrounding these are the group 2 neurons, which contain both epileptogenic and non-epileptogenic neurons. Thus Group 2 neurons are less epileptogenic overall. Interestingly, operant conditioning can reduce single cell firing in group 2 neurons. The normal neurons within the group 2 class are engaged by experimental enhancement of attention, and also during feeding times. These changes were related to a reduction in ictal events, suggesting the experimental manipulations prevented the recruitment of surrounding non-epileptogenic neurons into the spreading ictal focus. It is important to note that the manifestation of seizures in humans is not as simple as is suggested by the above animal models. Seizure activity is influenced in a number of distinct ways, including dependence on the site of seizure origin and intrinsic circuits supporting local seizure spread, and the extension of aberrant activity into motor regions to influence motor excitability and associated movements (Miller, 1992). Moreover in humans, emotional triggers may putatively interact with the epileptic focus via feedback of autonomic arousal, conveyed centrally by visceral afferent inputs.

## Autonomic activity in epilepsy and tourette syndrome

Autonomic activity is intrinsically linked to our emotional, mental, and physiological state. Emotional disturbances are accompanied by exaggerated autonomic activation. This is particularly the case with anxiety and stress, which coincides with enhanced sympathetic tone, and decreased vagus parasympathetic tone (Gianaros et al., 2012). Challenges to a patient's emotional, mental and physiological state similarly evokes an alteration in sympathovagal balance, and a state of physiological arousal, which is proposed to have a central role in the propensity for seizures in epilepsy (Nagai and Critchley, 2008), or tics in Tourette Syndrome (Wood et al., 2003). In turn, both the predisposition to and occurrence of seizures and tic activity can inversely impair autonomic regulation. Autonomic dysfunction leading to cardiac and respiratory arrest may be a significant contributor to Sudden Unexpected Death in Epilepsy (SUDEP) (Surges et al., 2009). Moreover, reduced heart rate variability, an index of diminished “cardioprotective” parasympathetic tone, is reported in patients with epilepsy compared to healthy controls (Persson et al., 2007), increasing vulnerability to cardiac arrest from arrhythmia. Thus in epilepsy, there is evidence for association between SUDEP and autonomic imbalance.

There is less evidence regarding the contribution of autonomic function in patients with Tourette Syndrome. An exaggerated initial heart rate acceleration during the Valsalva Manuever test (van Dijk et al., 1992) and a higher baseline heart rate and blood pressure (Tulen et al., 1998) in Tourette patients compared with healthy controls has been reported. Considering that genetic mutations cause both epilepsy and TS (Price et al., 1985; Robertson, 2000), a link between these mutations and autonomic function is of research interest.

Autonomic afferents are unmyelinated fibers originating from visceral organs, skin, and skeletal muscles. The information they carry is relevant to homeostasis (governed through both peripheral reflexes and central pathways) and contributes to autonomic, endocrine regulation, non-painful and painful visceral sensations (Janig, 2006). Visceral autonomic signals also shape emotional behaviors and arguably underpin emotional feeling states through conscious appraisal of changes signaled at a cortical level. Afferent neural pathways follow spinal and cranial nerves (principally the vagus nerve, Janig, 2006). Ascending spinal and vagus nerve afferents converge and terminate within the lower and upper brain stem, hypothalamus and thalamus. Vagal afferents enter the nucleus tract solitarius (NTS), then project through to the ventromedial part of the thalamus with spinal afferents and project to terminate as a primary interoceptive cortical representation within the insula cortex (Janig, 2006; Craig, 2009). Co-lateral thalamic projections also include to the primary and secondary somatosensory cortex (responsible for exteroception) and to the mid and anterior cingulate cortex (responsible for motivation behavior and interoception) (Janig, 2006; Craig, 2009). Importantly, this ascending viscerosensory information also interacts at multiple levels within the brainstem with both ascending and descending monoamine systems that mediate peripheral sympathetic activity and central arousal and attention (Critchley and Harrison, 2013). The reticular activating system, and its effects on thalamocortical information flow is particularly relevant, since it is speculated that thalamic sensitivity to the signaling of autonomic afferent arousal is a potent trigger of seizures and tics (Nagai et al., 2004c; Nagai and Critchley, 2008).

In an animal model of epilepsy (the Kainic acid model), progressive neuronal loss in the NTS and SUDEP like death is reported in rodents (Tolstykh and Cavazos, 2013). The loss of neurons in the NTS is directly attributed to frequent epileptic seizures (Tolstykh and Cavazos, 2013). Microinjection of a cholinergic agonist (carbachol) into the thalamus also elicits limbic and generalized seizures in rat (Mraovitch and Calando, 1999). The detailed observation of seizure behavior, and the timecourse of brain activity changes using identification of c-fos early gene activity revealed the immediate effect of an epileptogenic agent in regions of autonomic system such as parabrachical nucleus and hypothalamus, indicating the involvement of autonomic brain areas in the early stages of seizure activity. The recruitment of limbic areas and thalamo-cortical communication occurs later, around 60 min after injection (Mraovitch and Calando, 1999).

Animal models of Tourette Syndrome are rather fewer in number. However, alpha 1 adrenergic drug administration has been used to model tics (startle inhibition) in rat, and indicate a role of autonomic agents in the neural mechanism underlying Tourette Syndrome (Swerdlow et al., 2006).

**Figure 1:**
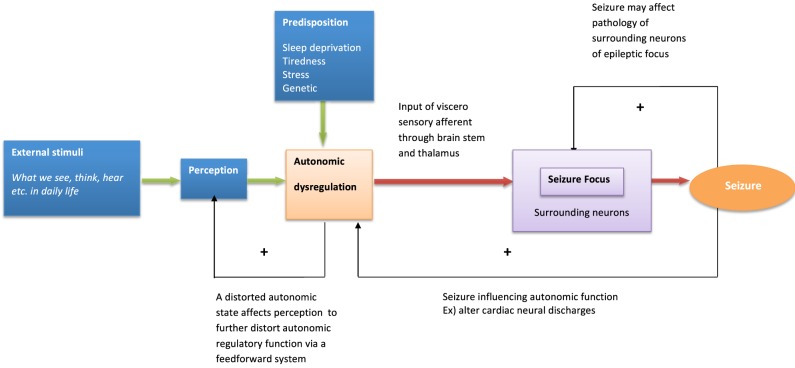
**Model of behavioral modulation and seizure generation**. The model describes psychological and neural interaction influencing seizure generation.

The following model illustrates how emotional stimuli may trigger epileptic seizures (Figure 1). Emotional disturbances can originate from sensory stimulation such as vision, hearing, and also from internal thoughts and memories. Whether or not such information predictably recruits autonomic activation depends on the intensity of stimulation, the significance of the timing and individual differences in sensitivity and salience to stimuli (Bauscein, 2006). Certain types of stimuli may preferentially engage autonomic systems (e.g., disturbing thoughts changing heart rate variability, Ottaviani et al., 2015). It is speculated that these visceral afferent influences affect neural excitability around seizure foci through effects mediated at the level of the reticular formation and thalamus. Indeed, in a model of complex partial seizures, a “Network Inhibition Hypothesis” proposes that impaired consciousness is attributed to suppression of midline subcortical structures (i.e., the reticular activation system). Spreading seizure activity affects the reticular activation system which leads to regional depression of frontoparietal association cortex known to be crucial for maintenance of consciousness (Blumenfeld, 2011). It is hypothesized that changes in thalamo-cortical regulation impacts on seizure threshold via physiological arousal and visceral feedback (Nagai, 2011). The typical emotional and behavioral triggers of seizures (stress, sleep deprivation, and fatigue) represent events that commonly disturb autonomic regulation. In cognitive behavioral terms, disrupted autonomic regulation in turn affects perception and cognition, potentially creating a vicious circle in a patient's emotional state. Looking at autonomic dysregulation from a different angle, seizure events themselves also affect autonomic activity: post ictal autonomic dysregulation is one candidate as a cause for SUDEP (Poh et al., 2012). Thus, autonomic dysregulation, reflecting interaction between underlying brain pathology (e.g., temporal lobe damage), seizure activity, the effect of anti-epileptic drugs and the psychosocial consequences of societal conditioning and stigma is inherently linked to the emotional resilience and vulnerability of patients. Patients with epilepsy have specific predisposing psychological and neural dynamics which underlie seizure generation (Ridsdale et al., 2013).

**Figure 2:**
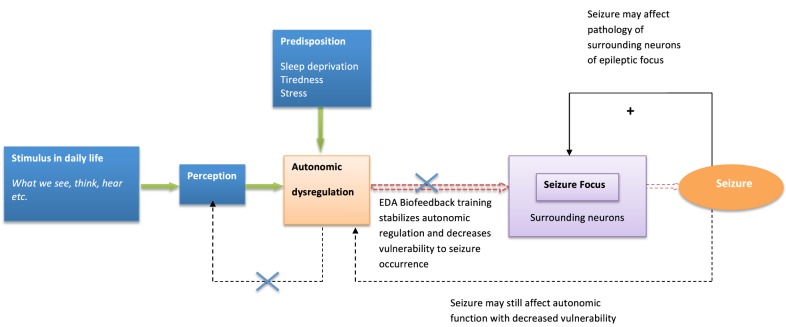
**Model of biofeedback intervention to behavioral modulation and seizure suppression**. The model describes biofeedback intervention to alter psychological and neural dynamics influencing seizure generation.

Similarly, psychological influences and autonomic dysfunction may also play a role in the expression of tics in TS, through thalamo-cortical-striatal dysregulation [known to underlie tic activity (Wang et al., 2011)]. Attempting to alter these complex dynamics by modifying physiological and psychological processes may represent a valuable treatment option (Figure 2).

## Modulation of autonomic activity as a treatment for epilepsy and tourette syndrome

While pharmacological approaches can be used to modulate autonomic activity, the enhancement of autonomic control (and its integration with other psychological and motoric functions) can be achieved clinically using electrodermal biofeedback (Nagai et al., 2004a,b). Biofeedback is a non-invasive intervention with which patients can be trained to control their physiological responses (Shwartz and Olson, 1995). A variety of physiological parameters are accessible for biofeedback however electrodermal activity (EDA) is typically an easy-to-implement autonomic parameter, which exclusively modulates sympathetic activity coupled to central psychological arousal. Electrodermal activity (EDA) reflects peripheral sympathetic nervous influences on sweat gland function, and is achieved by applying a small electrical current to the skin surface, to quantify changes in electrical resistance or conductance. The sympathetic innervation of sweat glands is uniquely cholinergic, hence unlike other effector organs with adrenergic/noradrenergic sympathetic innervation, activity is not sensitive to humoral monoaminergic levels. The measurement of EDA is a useful and sensitive physiological measurement of emotional and mental arousal, and is widely used in experimental conditions (Bauscein, 2006).

**Figure 3:**
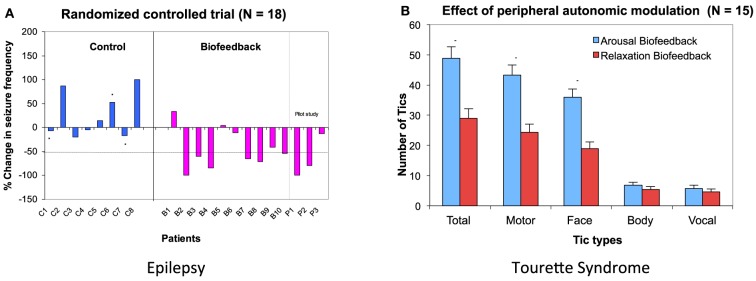
**Biofeedback intervention on Epilepsy and Tourette Syndrome**. **(A)** Data from a randomized controlled trial in epilepsy patients. Six patients out of 10 patients in biofeedback showed more than 50% seizure reduction after a month of EDA biofeedback intervention. Asterisk (^*^) show patients who dropped out in the middle of the training. **(B)** Effect of peripheral autonomic modulation in patients with Tourette Syndrome. EDA biofeedback was performed to increase and decrease skin conductivity. The number of tics occurring during 5 min of bio-feedback intervention are presented for each patient. (Nagai et al., 2004b, 2008, permission obtained).

EDA biofeedback as a treatment for epilepsy is a relatively new approach. However early evidence for its efficacy is promising and suggests great potential for this psychophysiological technique to become more available as an easy-to-implement and side-effect free treatment. Nagai et al. (2004a) conducted a preliminary randomized-controlled trial with 18 patients with drug resistant epilepsy. Patients were trained to increase their sympathetic activity through visual and auditory feedback of their EDA activity. The treatment consisted of a half an hour session, three times a week for 4 weeks. The results demonstrated significant seizure reduction in the biofeedback-active group, with over half these patient showing more than 50% seizure reduction after a month of EDA biofeedback training (Nagai et al., 2004a) (Figure 3A). Accompanying electroencephalography (EEG) recording showed baseline changes in an experimental measure of cortical excitability: the Contingent Negative Variation (CNV: an experimentally-induced slow cortical potential), suggesting EDA biofeedback induces long-term functional changes within neural networks underpinning cortical excitation (Nagai et al., 2009). This observed effect of EDA biofeedback was sustainable: a subset of patients voluntarily kept a seizure diary for a much longer term (over 3 years after treatment) and these records suggesting that the treatment may prompt long term neurobiological changes that maintain efficacy in seizure control (Nagai and Trimble, 2014). The clinical trial findings were recently replicated and showed similar results to the initial clinical trial (Micoulaud-Franchi et al., 2014).

The effect of EDA biofeedback training has also been examined in patients with Tourette Syndrome (Nagai et al., 2008, 2014). In contrast with the application of EDA arousal biofeedback in epilepsy, a preliminary study with patients with Tourette Syndrome showed that the reduction of patients' tics correlated with decreasing sympathetic activity (Nagai et al., 2008) (Figure 3B). However, the clinical application of EDA biofeedback for this patient group needs cautious consideration. Standard training procedures established for other conditions are much harder to implement in this patient group, due to an apparent difficulty in reducing sympathetic activity within this clinical population (Nagai et al., 2014). Interestingly, this further supports the notion of autonomic dysregulation in association with Tourette Syndrome. The presence of intermittent tics, and arguably the preceding “urge to tic,” make it particularly difficult for patients to successfully learn, perform and sustain EDA “relaxation” biofeedback with typical training schedules (Nagai et al., 2014). However, tic activity is reduced over short periods of EDA biofeedback that suppress sympathetic activity (Nagai et al., 2009), and this motivates further exploration for translation into an effective intervention.

## Neural mechanisms of autonomic control in suppression of seizures and tics

EDA biofeedback is an easy-to-implement, potent and reliable means of modulating autonomic activity. However, the detailed neural mechanisms by which seizures are suppressed by EDA biofeedback are still under investigation. A plausible explanation of the clinical effect of EDA in epilepsy includes changes in thalamo-cortical regulation leading to an increased seizure threshold across the cortex. The modulation of cortical potentials by EDA biofeedback may be important in reducing seizure susceptibility in patients. The association of seizure activity with slow cortical potentials is reinforced by the observation that paroxysmal ictal activity is often accompanied by a negative DC potential shift (direct current shift toward negative amplitude) in patients (Chatrian et al., 1968). In animal models, a localized cortical negative potential is also produced by chemical induction (generalized seizure elicited by intraperitoneal or interavenous pentylenetetrazol) of focal epilepsy (Casper and Speckman, 1972). Experimentally, slow cortical potentials can be induced using a behavioral paradigm, a forewarned reaction time task, that generates the CNV. A negative electrical DC shift is measurable over the midline cortical area during the anticipatory period between the presentation of a warning cue (to “get-ready”) and an imperative stimulus (to respond). The cortical excitability over this period is accompanied by enhanced central arousal and action-related preparatory autonomic adjustments. Changes in peripheral EDA activity modulate CNV amplitude, whereby the magnitude of the slow cortical potential was inversely related to the state of EDA measures of sympathetic arousal (Nagai et al., 2004b). This observation in fact motivated the therapeutic application of EDA “arousal” biofeedback (i.e., training to increase sympathetic EDA) as a means to decrease cortical excitation in epilepsy.

**Figure 4:**
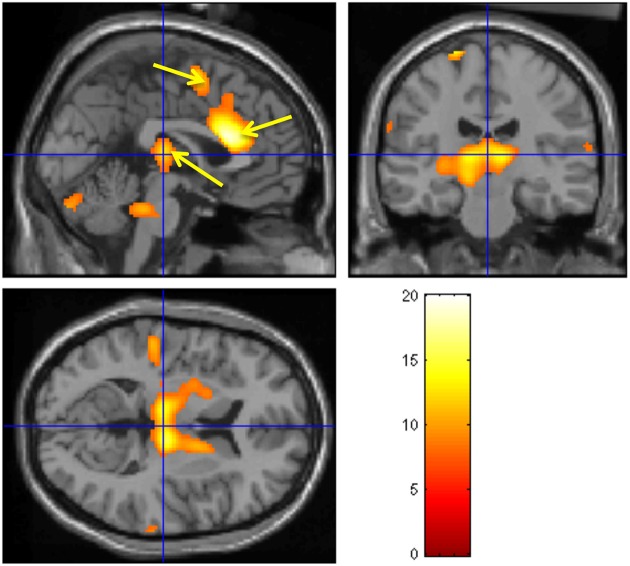
**Brain activity modulated by the CNV amplitude**. Neural activities associated with CNV generation. The observed regions during the CNV task include bilateral thalamus, ante- rior cingulate, SMA, pons, and cerebellum. A fixed-effect analysis was used (*p* < 0.05, cor- rected). (Nagai et al., 2004c, permission obtained).

**Figure 5:**
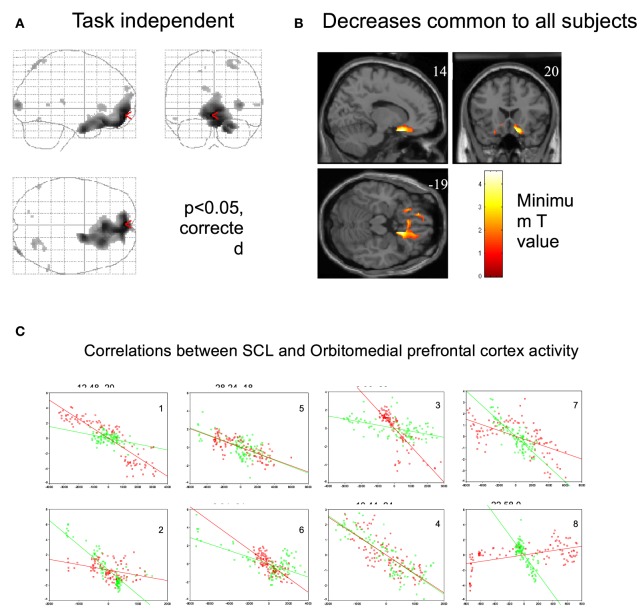
**Regional brain activity associated with decrease in skin conductance level**. (Nagai et al., 2004d, permission obtained). **(A)** Regional brain activity related to task-independent decreases in skin conductance level (SCL). Decreases in SCL were associated with increased activity in VMPFC and OFC. (*P* < 0.05, corrected). During the EDA biofeedback, activity in VMPFC and OFC is decreased. **(B)** Regional brain activity associated with decreases in skin conductance level common across all subjects (*P* < 0.05, corrected). Common brain activity was found in VMPFC and OFC. **(C)** Plots of individual subjects data showing correlations between skin conductance and ventromedial and orbitofrontal BOLD activity during biofeedback relaxation (green dots) and arousal tasks (red dots). There was a significant negative correlation between adjusted VMPFC and OFC BOLD responses and task-independent SCL activity in the eight subjects.

A neuroimaging study was conducted in healthy participants to identify brain regions involved in the generation of the CNV: on a trial-by-trial basis, CNV correlated with enhancement of activity within the thalamus, anterior cingulate and supplementary motor area (SMA), revealing the thalmo-cortical network ultimately responsible for generation of the CNV (Nagai et al., 2004c) (Figure 4). A parallel neuroimaging study also revealed brain regions engaged during EDA biofeedback. Activation within the ventro-medial prefrontal cortex (VMPFC) and adjacent medical orbitofrontal cortex (OFC) were linearly, yet inversely, correlated with the level of skin conductance during EDA biofeedback (Nagai et al., 2004d) (Figure 5). Thus increased EDA sympathetic arousal following biofeedback task performance resulted in a decrease in the activation of VMPFC and medial OFC, while biofeedback-induced decreases in EDA levels resulted in an enhanced activation of these regions. Interestingly, the VMPFC and medial OFC are both involved in viscerosensory and form an important hub within the default mode network, which deactivates during states of behavioral arousal. Arguably the functional manipulation of the reactivity of this brain region over a month of EDA biofeedback training may, for patients with epilepsy, enhance the degree to which thalamo-cortical excitability, and its gating by the ascending reticular activating system, is suppressed during behavioral arousal. The functional impairment of this network, reported in patients with epilepsy, may be normalized, ultimately leading to decreased vulnerability to internal and external seizure triggers. In the case of Tourette Syndrome and related tic disorders, the training to diminish levels of physiological arousal may help increase the threshold for tics through the interaction between motor pathways and autonomic central command. Alternatively, the relationship with premonitory urges and compulsive behavior may be an important mechanism: damage to the viscerosensory insula cortex is observed to inhibit addictive urges and compulsions to smoke (Gray and Critchley, 2007; Naqvi and Bechara, 2009). Arguably, biofeedback “relaxation” training can decrease viscerosensory arousal signals that underlie or reinforce the feelings of urge and compulsions. This effect may be mediated by the cortical representation of the arousal state within default mode and salience networks, which in turn influence motor excitability and ascending monoaminergic pathways. While this provides a neurobiological model, there needs to be further work in extending and translating current knowledge of autonomic interaction with tics.

## Conclusion

The role of autonomic nervous system activity in many neurological conditions, including movement disorders, has not been systematically investigated, yet compelling examples exist, some presented here, of how autonomic function bridges psychological and physical states to influence symptom expression. In the current manuscript, a general principle is illustrated for two neurological conditions, epilepsy and Tourette Syndrome. However, the insights into the role of autonomic function in the expression of clinical symptoms and presentation may usefully extend to other neurological and neuropsychiatric disorders. These observations allow us to begin to construct theoretical models for experimental and translational science, ultimately to provide novel or individualized interventions to improve patients' physiological and psychological condition. It is proposed that autonomic biofeedback is a valuable therapeutic tool, the potential of which has yet to be fully realized. In a wider clinical trial, it is also important to investigate different subtypes of seizures and tics, as the effectiveness of autonomic modulation, and vulnerability to emotional challenges, may present differently. It is anticipated that biofeedback can be used to enhance the emotional and physiological stability of patients with direct beneficial consequences for neurological symptoms.

### Conflict of interest statement

Ultrasis Plc owns a patent arising from Yoko Nagai's work, from which Yoko Nagai may potentially benefit.
